# Bile Acid Supplementation Improves Murine Pancreatitis in Association With the Gut Microbiota

**DOI:** 10.3389/fphys.2020.00650

**Published:** 2020-06-16

**Authors:** You-Dong Wan, Rui-Xue Zhu, Xin-Ting Pan, Tong-Wen Sun

**Affiliations:** ^1^Department of Emergency Intensive Care Unit, The Affiliated Hospital of Qingdao University, Qingdao, China; ^2^Health Management Center, The Affiliated Hospital of Qingdao University, Qingdao, China; ^3^Department of Integrated Intensive Care Unit, The First Affiliated Hospital of Zhengzhou University, Zhengzhou, China

**Keywords:** pancreatitis, microbiota, bile acids, 16S rDNA, intestinal microbiota

## Abstract

Disorders of bile acids (BAs) are closely related to the development of liver and intestinal diseases, including acute pancreatitis (AP). However, the mechanism underlying the involvement of BAs in AP development remains unclear. We used intraperitoneal injection of cerulein to construct AP mouse models. These mice had significantly reduced tauroursodeoxycholic acid (TUDCA) and an imbalance of intestinal microbiota, based on 16S rDNA gene sequencing. To explore the role of AP-induced intestinal microbiota changes in the development of AP, we transplanted the stool obtained from AP mice to antibiotic-treated, microbiota-depleted healthy mice. Microbiota-depleted mice presented injury to the intestinal barrier function and pancreas. Additionally, microbiota depletion reduced AP-associated pancreatic injury. This indicated that the gut microbiota may worsen AP. As TUDCA was deficient in AP mice, we gavaged AP mice with it, and evaluated subsequent expression changes in the bile acid signaling receptors farnesoid-x-receptor (FXR) and its target gene fibroblast growth factor (FGF) 15. These were downregulated, and pancreatic and intestinal barrier function injury were mitigated. The gut microbiota is known to regulate bile acid production and signaling, and our analysis of changes to the gut microbiota in AP indicated that *Lactobacilli* may be the key contributors of TUDCA. Taken together, our study shows that supplementation with BAs could reduce pancreatic and intestinal injury, and that this effect may be associated with the gut microbiota.

## Introduction

An important component of bile participating in fat metabolism, BAs can also act as signaling molecules by interacting with cell membranes and nuclear receptors, and play important roles in glucose and lipid metabolism and energy homeostasis (Martinot et al., 2017). Recent studies have found that dysregulated BAs are closely associated with hepatopathy, for example, steatohepatitis, hepatocellular carcinoma, and intestinal diseases such as colorectal cancer (Arab et al., 2017; Joyce and Gahan, 2017). BAs circulating in the gastrointestinal tract are important messengers linking intestinal microecology and intestinal diseases. Specifically, intestinal microbes induce proportional and structural changes to bile acid composition, thereby producing biological effects (Ramirez-Perez et al., 2017). Two key receptors of BAs are the Takeda G-protein coupled receptor clone 5 (TGR5) and the farnesoid-X-Receptor (FXR) (Martinot et al., 2017). FXR is a ligand-dependent transcription factor, belonging to the nuclear receptor superfamily, and is mainly expressed in the liver, intestine, kidney, and adrenals. FXR activates fibroblast growth factor (FGF)19 in humans and FGF15 in mice (Kliewer and Mangelsdorf, 2015). Activation of bile acid receptors can further activate several specific signaling pathways including lipid metabolism, the immune system, signal transduction, and others (Kliewer and Mangelsdorf, 2015).

Acute pancreatitis (AP) is an acute digestive disease characterized by acute upper abdominal pain, elevated serum amylase, exudation of pancreatic edema, and inflammation, with a mortality rate as high as 10–30% (Tenner et al., 2013). Recent studies indicate that intestinal microbiota participate in the occurrence and development of pancreatitis (Tan et al., 2015; Signoretti et al., 2017). Our group has previously reported dramatically changed intestinal microbiota in rats with pancreatitis, and we were able to improve the intestinal microbiota imbalance of theses rats with anti-inflammatory therapy (Wan et al., 2019). Evidence also indicates that bile acids (BAs) are involved in the development of AP, but the exact mechanisms underlying this association are unclear, especially in non-biliary pancreatitis (Hegyi et al., 2018).

As gut microbiota regulates BA production and signaling, we assumed that AP-associated gut microbiota changes could result in alterations of bile acid profiles, activating bile acid-FXR-FGF15 signaling, and resulting in pancreatic and intestinal injury. Therefore, we hypothesized that adjusting BA levels may treat AP. In order to answer these questions, we undertook three principal experiments with murine models of AP. These examined microbiota composition and BA levels in AP, the effects of microbiota deletion and transplant, and the effects of TUDCA supplementation.

## Materials and Methods

### Murine AP Model

This study was carried out in strict accordance with the laboratory animal management guidelines of Qingdao University, and approved by the ethics committee of Qingdao University animal experiments. Healthy male C57BL/6 mice weighing between 25 and 30 g were supplied by the Laboratory Animal Center of Qingdao University (Qingdao, China). We constructed the AP model following the method of Ding et al. (2003) Briefly, mice in the AP group were given ten hourly intraperitoneal injections of a supramaximal dose of cerulein (Sigma-Aldrich, St. Louis, MO, United States). Lipopolysaccharide (Sigma-Aldrich, St. Louis, MO, United States) was administered by intraperitoneal injection immediately after the 10th injection of cerulein. Mice intraperitoneally injected with saline were used as controls.

#### Experimental 1: Analysis of Gut Microbiota and BA Levels in AP

Thirty C57BL/6 mice were randomly divided into a sham-operated group (the SO group) and an AP group, with 15 mice in each group. Mice were sacrificed at 24 h after AP induction. Pancreatic and ileal tissues were harvested individually and fixed in 40 g/L formaldehyde. Samples were then embedded in paraffin and continually sectioned. We collected fresh stool samples before the mice were sacrificed and stored them at −80°C before subjecting them to analyses of bile acid levels and microbiota (Sections “Analysis of Intestinal Microbiota” and “Bile Acid Analysis”). Blood was collected by cardiac puncture and centrifuged at 15 000 rpm for 15 min, followed by testing for trypsin and inflammatory cytokines.

#### Experimental 2: Effects of Gut Microbiota Deletion and Transplant

To further explore the role intestinal microbiota plays during AP, we treated healthy mice with broad-spectrum antibiotics for 21 consecutive days to deplete intestinal bacteria, thus creating gut microbiota-depleted (GMD) mice. Validation of the gut bacterial depletion was performed through a fecal culture, in which brain-heart infusion agar plates were incubated anaerobically at 37°C for 48 h. Fecal samples were harvested before and after antibiotic treatment, and stool supernatant from AP mice was fed by gavage to GMD mice for 3 days to create fecal microbiota-transplanted (FMT) mice. The bacterial composition between AP and FMT mice was compared using 16S rDNA gene sequencing to confirm successful fecal transplant (Section “Analysis of Intestinal Microbiota”). Further details of the GMD and FMT model mice are described in Supplementary Material 1. In brief, 60 C57BL/6 mice were randomly divided into four groups of 15 mice as follows: an SO group, an AP group, an GMD + AP group, and an FMT group. Pancreatic tissues, blood, and stool samples were collected from all mice.

#### Experimental 3: Effects of TUDCA Supplementation

In the first experiment, TUDCA was found to be reduced in AP. We therefore fed AP mice a diet of standard laboratory chow supplemented with 0.4% TUDCA (Cayman Chemicals, Ann Arbor, MI, United States) to explore the effect of bile acid supplementation on AP (Supplementary Figure S3). As BAs and their conjugated forms were identified as FXR ligands, we analyzed the bile acid-FXR-FGF15 signaling axis using RT-PCR (Section “Real-Time PCR Analysis”). In this experiment, 60 C57BL/6 mice were randomly divided into five groups of 12 as follows: an SO group, an AP group, an AP + TUDCA pancreatitis group, a GMD pancreatitis group, and a GMD + TUDCA pancreatitis group.

### Analysis of Intestinal Microbiota

Samples were analyzed by 16S rDNA gene sequencing, the details of which we have reported previously (Wan et al., 2019). Operational taxonomic units (OTUs) that reached 97% similarity and Shannon index were used for α-diversity estimations. Non-metric multidimensional scaling methods were conducted to visualize differences between two groups. Linear discriminant analysis was used to explore principal differences between types of bacteria. Details of 16S rDNA gene sequencing and our intestinal microbiota functional annotation are given in Supplementary Material 1.

### Bile Acid Analysis

Bile acid levels in feces were quantitatively measured by ultra-performance liquid chromatography triple quadrupole mass spectrometry (UPLC-TQMS) according to the following protocol. The fecal samples were extracted with methanol and the supernatant was transferred and vacuum-dried. UPLC-MS raw data obtained with negative mode were analyzed using TargetLynx applications manager version 4.1 (Waters Corp., Milford, MA, United States) to obtain calibration equations and the quantitative concentration of each bile acid in the samples. For details, see Supplementary Material 1.

### Histological Evaluation and Measurement of Amylase D-Lactate Inflammatory Cytokines

Pancreas and distal ileum samples were stained with hematoxylin and eosin (HE), and examined and scored with a published system for grading of intestinal tissue injury (Chiu et al., 1970). Amylase activity, D-lactate level, and diamine oxidase activity in serum were measured using enzyme assay kits (Shanghai Hengfei Bioscience, China). The levels of IL-1β, TNF-α, and IL-6 were measured using an ELISA kit following the manufacturer’s instructions (LMAIBio Biotech, China).

### Real-Time PCR Analysis

Bile acid-FXR-FGF15 signaling axis was assessed using quantitative PCR. Intestinal mucosa scraped from the ileum were frozen in liquid nitrogen and stored at −80°C. The expression levels of the genes FXR, SHP, and FGF15 were tested. A standard phenol-chloroform extraction was performed to isolate total RNA from frozen tissues with Trizol reagent. Synthesis of cDNA was performed from 2 μg of total RNA with a Reverse Transcription Kit (Shanghai Hengfei Bioscience, China). The real-time PCR primer sequences are listed in Supplementary Material 1.

### Statistical Analysis

Values are expressed as the mean ± SEM. Significant differences between two groups were evaluated with a two-tailed, unpaired Student’s *t*-test, or Mann–Whitney *U* test for samples that were not normally distributed. Multiple groups were analyzed by one-way or two-way ANOVA followed by Bonferroni or Dunnett’s multiple comparison test. Correlation analyses involving the gut microbiome and bile acid metabolism were performed using the non-parametric Spearman’s test. Data were subjected to statistical analysis using SPSS 15 software (SPSS, Chicago, IL, United States); *P* < 0.05 was considered statistically significant.

## Results

### Changes to Gut Microbiota and Bile Acid Metabolism in AP Mice

**FIGURE 1:**
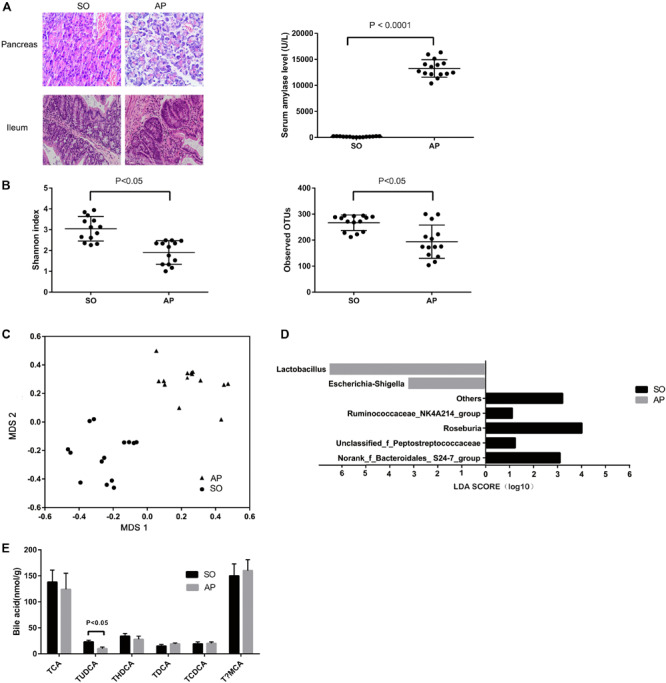
Gut microbiota and bile acid metabolism changes in AP mice. **(A)** Histological analysis of the pancreas and ileum, and analysis of serum amylase levels in sham-operated (SO) and acute pancreatitis (AP). **(B)** The Shannon diversity index and numbers of observed OTUs (a-diversity) are different between AP and SO. **(C)** Non-metric multidimensional scaling method showing a definite shift in gut microbiota composition between AP and SO. The horizontal axis represents the first dimension and the vertical axis represents the second dimension. **(D)** Linear discriminant analysis scores for the bacterial taxa differentially abundant between acute pancreatitis and the sham-operated group. Only the taxa having a *p* value < 0.01 and LDA > 2.0 were shown. **(E)** Stool bile acid levels in the SO and AP groups.

Acute pancreatitis was induced by intraperitoneal injections of cerulein and was assessed based on amylase quantification and histopathological changes in the pancreatic tissue. Pancreatic inflammatory cell infiltration, hemorrhage, ileal edema, and shortened villi were observed in the AP group (Figure 1A). Compared with the SO group, the levels of amylase in serum were significantly increased in the AP group (*P* < 0.05) (Figure 1A). The above results indicated that the AP model was established successfully. To identify AP-induced changes to the composition of the gut microbiota, we conducted 16S rDNA amplicon sequencing. The Shannon diversity index and numbers of observed OTUs (a-diversity) of gut microbiota were remarkably decreased after AP induction (Figure 1B). Our non-metric multidimensional scaling method showed that the gut microbiota composition was substantially reshaped in the AP group (Figure 1C). Linear discrimination analysis coupled with effect size analysis revealed significant increases of *Lactobacillus* and *Escherichia-Shigella* and substantial reductions of *Roseburia, Ruminococcaceae_NK4A214_group, norank_f_Bacteroidales_ S24-7_group*, and *unclassified_f_Peptostreptococcaceae* in AP compared with SO (Figure 1D). To characterize the functional alterations of the gut microbiota in AP, the relative abundances of KEGG pathways predicted by PICRUSt were calculated based on the 16S rRNA sequencing data. Multiple KEGG categories were dysregulated in AP compared to SO. There was significant enhancement of the pathways for infectious diseases, metabolism of terpenoids and polyketides, immune system diseases, and signal transduction, and significant weakening of genetic information processing, the circulatory system, transcription, and digestive system pathways (Supplementary Figure S1A).

We used UPLC-TQMS metabolite profiling to quantitate bile acid levels in the stool. The level of TUDCA was significantly decreased in the AP group (Figure 1E). Total bile acid levels remained unchanged, but noticeable decreases in ratios of conjugated to unconjugated BAs were observed. There was no difference in the ratio of 12α-OH to non-12α-OH BAs (Supplementary Figure S1B). Altogether, these data suggested that AP changed the gut microbiota and bile acid metabolism in mice.

### AP-Associated Changes to the Gut Microbiota Aggravate AP

**FIGURE 2:**
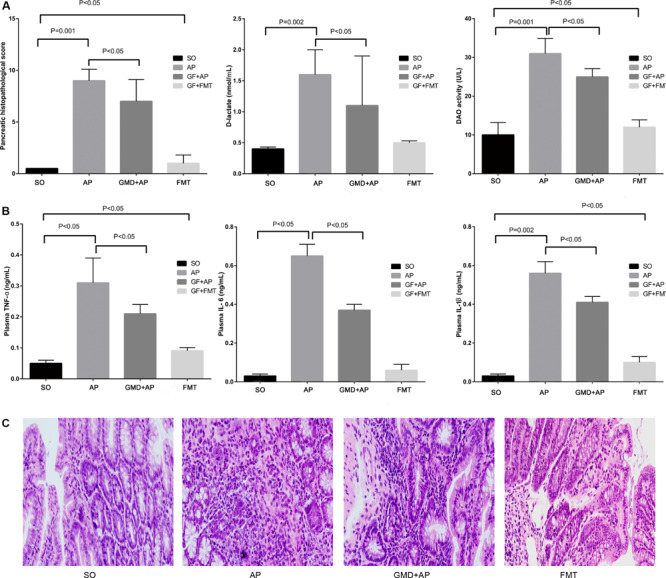
Acute pancreatitis gut microbiota worsen pancreatitis. **(A)** Changes to pancreatic histopathology, plasma D-lactate, and diamine oxidase between sham-operated (SO), acute pancreatitis (AP), gut microbiota-depleted (GMD), and fecal microbiota transplantation (FMT) mice. Stool supernatant from AP mice was fed by gavage to GMD mice for 3 days to create fecal microbiota-transplanted (FMT) mice. **(B)** Changes to levels of intestinal inflammatory cytokines (tumor necrosis factor α, IL-1β, and IL-6). **(C)** Histological analysis of the ileum in the SO, AP, GMD + AP, and FMT groups.

To explore the role of AP-induced intestinal microbiota changes in the development of AP, we used four groups of mice: SO, AP, GMD + AP, and GMD mice given fecal transplant from AP mice (FMT). Pancreatic histopathological changes were mitigated in the GMD + AP group compared to the AP group (Figure 2A). Based on plasma D-lactate and diamine oxidase levels, intestinal barrier function injury was mitigated in GMD + AP (Figure 2A). Plasma levels of TNF-a, IL-1β, and IL-6 were significantly decreased in the GMD + AP group (Figure 2B). Interestingly, in the FMT group, mildly aggravated ileum histology, intestinal barrier function, and plasma inflammation were found (Figure 2C). Altogether, these data suggested that AP gut microbiota aggravate AP.

### Bile Acid Supplementation Improves Pancreatitis

**FIGURE 3:**
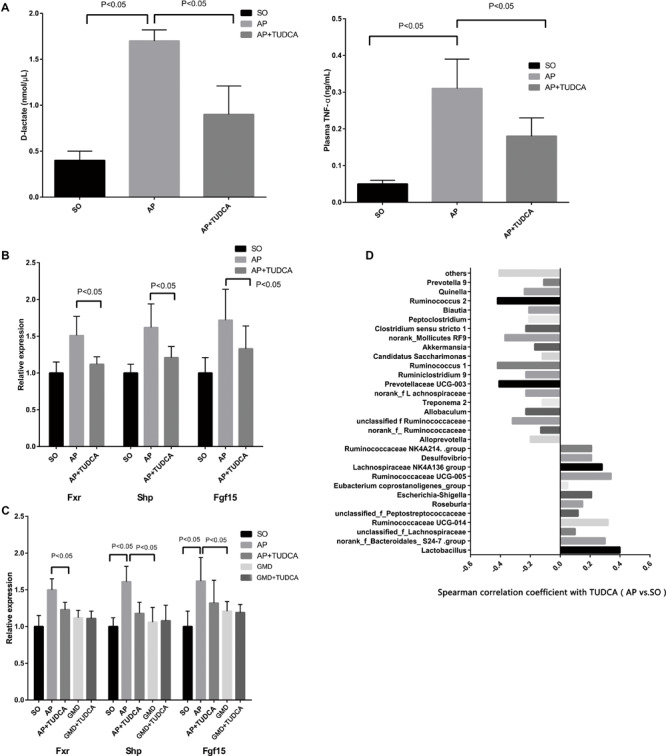
Tauroursodeoxycholic acid (TUDCA) has therapeutic effects on acute pancreatitis associated with gut microbiota. **(A)** Changes of D-lactate and tumor necrosis factor αlevels between the three groups. **(B)** Relative expression of intestinal FXR mRNA and its target gene mRNAs in mice. **(C)** Relative expression of intestinal Fxr, Shp, and Fgf15 mRNAs in mice. **(D)** Spearman correlations between the most abundant genera and TUDCA.

To explore the effect of bile acid supplementation, we gave AP mice TUDCA, in view of its observed reduction in AP. This resulted in significantly decreased plasma D-lactate, diamine oxidase, TNF-α, IL-1β, and IL-6 levels, indicating improved intestinal barrier function and ameliorated plasma inflammation during AP (Figure 3A and Supplementary Figure S2). Although BAs and their conjugated forms have been identified as FXR or TGR5 ligands, a previous study demonstrated that TUDCA has no effect on TGR5 activity (Sun et al., 2018). However, activation of the FXR-small heterodimer partner (SHP) inhibits bile acid synthesis. Therefore, we evaluated expression of FXR target gene mRNAs including FXR, FGF15, and SHP. We found that the intestinal FXR-FGF15 axis was downregulated with TUDCA supplementation in AP mice (Figure 3B). To investigate whether the gut microbiota was involved in the inhibition of intestinal FXR signaling by bile acid, GMD mice were used. When gut microbiota was depleted in AP mice, the severity of pancreatitis decreased (Figure 2, GMD + AP group) and the FXR-FGF15 axis was downregulated (Figure 3C). Similarly, when we gave TUDCA to AP mice, pancreatitis was mitigated (Figure 3A), and the FXR-FGF15 axis was downregulated (Figure 3B). However, the significant further inhibition of FXR signaling was not observed in the GMD plus TUDCA group (Figure 3C). The gut microbiota is known to regulate bile acid production and signaling (Ramirez-Perez et al., 2017), the effect of gut microbiota in decreased severity of AP may through regulates bile acid levels. Correlating bacterial composition with TUDCA in AP vs. SO, we found positive associations for *Lactobacillus* (*P* < 0.05), and some other AP-enriched bacteria including *Ruminococcus 2, Prevotellaceae UCG-003*, and *Ruminococcus 1* (Figure 3D). Altogether, these data suggest that gut microbiota may participate in pancreatitis, and that bile acid supplementation can mitigate the disease.

## Discussion

The invasion of bile acid into the pancreatic duct, caused by bile reflux, has traditionally been considered part of the pathogenesis of acute biliary pancreatitis. However, the role and mechanism of bile acid in non-biliary pancreatitis is still unclear. In this study, the level of TUDCA was reduced in mice with non-biliary pancreatitis. With TUDCA supplementation, the AP mice showed improved pancreatic and intestinal injury and decreased plasma inflammation. Further study indicated that gut microbiota may participate in pancreatitis.

Tauroursodeoxycholic acid is a non-toxic taurine conjugate form of ursodeoxycholic acid, which is an endogenously produced hydrophilic bile acid. Several studies have shown its potential for treating liver diseases (Carey et al., 2015), and possible mechanisms underlying this include prevention of cell death by stabilization of the cell membranes, inhibition of apoptosis, and upregulation of survival pathways (Schoemaker et al., 2004). However, to our knowledge, the effects of TUDCA on AP have not been investigated previously. At present, the main AP treatments include fluid resuscitation, nutritional support, and infection prevention (Vege et al., 2018).

In view of evidence that the gut microbiota regulates bile acid production and signaling (Ramirez-Perez et al., 2017), we also investigated their relationship with AP and BAs. Our fecal transplantation experiment showed that the gut microbiota from AP mice were harmful to the intestinal function of healthy mice. Moreover, although deletion of gut microbiota and supplementation with TUDCA were both useful for the treatment of AP, our third experiment showed that deletion of gut microbiota plus TUDCA supplementation resulted in no additional therapeutic effect, with gene expression analysis revealing that gut microbiota may participate in inhibition of intestinal FXR signaling via TUDCA.

Several studies have explored the association between gut microbiota and AP by intestinal gene sequencing. Zhu et al. (2019) found that increased capacity for bacterial invasion of epithelial cells in AP correlated closely with the abundance of *Escherichia-Shigella* in fecal samples from 165 adults. Zheng et al. (2019) showed that commensal *Escherichia coli* MG1655 increases TLR4/MyD88/p38 MAPK and ERS signaling-induced intestinal epithelial injury and aggravates AP in rats. These authors inferred gut microbiota dysbiosis in AP and tried to explain it from different perspectives. Similar to their results, our study also found dysbiosis of the gut microbiota in AP, and the altered BA metabolism we observed is a novel potential mechanism.

The initial and major sites of injury in AP are the pancreas and intestines. BAs are critical components of the gastrointestinal tract that link the gut microbiota to hepatic and intestinal metabolism. The ability of the gut microbiota to biotransform intestinal BAs into their unconjugated forms is central to the metabolic homeostasis of the gastrointestinal tract (Ramirez-Perez et al., 2017). The main bacterial genera of gut microbiota involved in bile acid metabolism include *Bacteroides, Clostridium, Lactobacillus, Bifidobacterium*, and *Listeria* (Gerard, 2013). Pancreatitis can damage the intestinal micro-environment, and thus change BA metabolism. Disruption of bile acid-microbiota crosstalk can promote inflammation, organ injury, and gastrointestinal disease phenotype, which can contribute to the development of gastrointestinal cancers, including colorectal cancer and hepatocellular carcinoma (Yoshimoto et al., 2013). In our study, disruption of bile acid-microbiota crosstalk manifested as decreases in a variety of bacterial species and their proportions, and functional alterations of the gut microbiota were reflected by decreased ratios of conjugated to unconjugated BAs, and TUDCA deficiency. These changes were associated with further injury to the pancreas and intestines. In a study by Sun et al. (2018), TUDCA was confirmed as an FXR antagonist *in vitro* and *in vivo*, and our study yielded similar results.

We found that TUDCA supplementation could improve bile acid-FXR-FGF15 signaling and reduce pancreatic and intestinal injury in AP, but the mechanism remains unclear. Qin et al. (2005) gave C57BL/6 mice a chow diet supplemented with increasing concentrations of BAs for 5 days. They found that FXR signaling was activated and dose-dependent induction levels of TNF-alpha, VCAM-1, ICAM-1, and SAA-2 mRNA were observed. This suggested that anti-inflammatory activity might be a possible mechanism by which the activation of FXR signaling mitigated pancreatitis. In a study by Seyhun et al. (2011), AP was induced in Wistar rats using cerulein, with or without TUDCA treatment. Similarly to our study, they found that TUDCA treatment reduced intracellular trypsin activation, edema formation, and cell damage. They also found TUDCA prevented cerulein-induced chaperone binding protein upregulation, and reduced X-box binding protein-1 splicing, myeloperoxidase, caspase 12 and 3 activation, and endoplasmic reticulum stress. Intermittent hypoxia is known to cause apoptosis in pancreatic β-cells, Song et al. (2018) established animal and cell models of intermittent hypoxia and found that inhibition of endoplasmic reticulum stress with TUDCA partially blocked intermittent hypoxia-induced autophagy. Thus, inhibition of endoplasmic reticulum stress and anti-inflammatory effects are possible mechanisms by which TUDCA mitigates pancreatitis.

Several limitations of this study should be addressed. First, the effect of BAs on changes to the FXR-FGF 15 axis we observed need further verification with intestinal-specific Fxr knockout mice and floxed control mice, which is an aim of our future work. Second, we showed that *Lactobacillus* was positively correlated with TUDCA, but the evidence was quite weak. The mechanism by which microbial dysbiosis causes altered bile acid levels during AP should be studied further. Previous work (Jia et al., 2018) has suggested several possible mechanisms, including BA deconjugation with *Lactobacillus*, BA esterification, and the action of bile salt hydrolase. Third, we could not identify a significant number of bacterial species because of technological limitations; with advances in sequencing technology, more gut bacteria relevant to pancreatitis may be detected.

In conclusion, BA supplementation could improve bile acid-FXR-FGF15 signaling, and reduce pancreatic and intestinal injury in AP, and this effect may be associated with the gut microbiota.

## Data Availability Statement

The 16S sequencing data has been deposited into figshare (DOI: 10.6084/m9.figshare.12326294).

## Ethics Statement

The animal study was reviewed and approved by the Ethics Committee of Qingdao University animal experiments.

## Author Contributions

Y-DW wrote the manuscript. T-WS revised the manuscript. Y-DW, R-XZ, and X-TP performed the data analysis. Y-DW and T-WS performed the data collection.

## Conflict of Interest

The authors declare that the research was conducted in the absence of any commercial or financial relationships that could be construed as a potential conflict of interest.

## References

[B1] Arab J. P. Karpen S. J. Dawson P. A. Arrese M. Trauner M. (2017). Bile acids and nonalcoholic fatty liver disease: molecular insights and therapeutic perspectives. *Hepatology* 65 350–362. 10.1002/hep.28709 27358174 PMC5191969

[B2] Carey E. J. Ali A. H. Lindor K. D. (2015). Primary biliary cirrhosis. *Lancet* 386 1565–1575. 26364546 10.1016/S0140-6736(15)00154-3

[B3] Chiu C. J. McArdle A. H. Brown R. Scott H. J. Gurd F. N. (1970). Intestinal mucosal lesion in low-flow states. I. A morphological, hemodynamic, and metabolic reappraisal. *Arch. Surg.* 101 478–483. 5457245 10.1001/archsurg.1970.01340280030009

[B4] Ding S. P. Li J. C. Jin C. (2003). A mouse model of severe acute pancreatitis induced with caerulein and lipopolysaccharide. *World J. Gastroenterol.* 9 584–589. 12632523 10.3748/wjg.v9.i3.584 PMC4621587

[B5] Gerard P. (2013). Metabolism of cholesterol and bile acids by the gut microbiota. *Pathogens* 3 14–24. 10.3390/pathogens3010014 25437605 PMC4235735

[B6] Hegyi P. Maleth J. Walters J. R. Hofmann A. F. Keely S. J. (2018). Guts and gall: bile acids in regulation of intestinal epithelial function in health and disease. *Physiol. Rev.* 98 1983–2023. 10.1152/physrev.00054.2017 30067158

[B7] Jia W. Xie G. Jia W. (2018). Bile acid-microbiota crosstalk in gastrointestinal inflammation and carcinogenesis. *Nat. Rev. Gastroenterol. Hepatol.* 15 111–128. 10.1038/nrgastro.2017.119 29018272 PMC5899973

[B8] Joyce S. A. Gahan C. G. (2017). Disease-associated changes in bile acid profiles and links to altered gut microbiota. *Dig. Dis.* 35 169–177. 10.1159/000450907 28249284

[B9] Kliewer S. A. Mangelsdorf D. J. (2015). Bile acids as hormones: the FXR-FGF15/19 pathway. *Dig. Dis.* 33 327–331. 10.1159/000371670 26045265 PMC4465534

[B10] Martinot E. Sedes L. Baptissart M. Lobaccaro J. M. Caira F. (2017). Bile acids and their receptors. *Mol. Aspects Med.* 56 2–9. 28153453 10.1016/j.mam.2017.01.006

[B11] Qin P. Borges-Marcucci L. A. Evans M. J. Harnish D. C. (2005). Bile acid signaling through FXR induces intracellular adhesion molecule-1 expression in mouse liver and human hepatocytes. *Am. J. Physiol. Gastrointest. Liver Physiol.* 289 G267–G273. 15817812 10.1152/ajpgi.00043.2005

[B12] Ramirez-Perez O. Cruz-Ramon V. Chinchilla-Lopez P. Mendez-Sanchez N. (2017). The role of the gut microbiota in bile acid metabolism. *Ann. Hepatol.* 16 s15–s20. 10.5604/01.3001.0010.5494 29080339

[B13] Schoemaker M. H. Conde D. L. R. L. Buist-Homan M. Vrenken T. E. Havinga R. (2004). Tauroursodeoxycholic acid protects rat hepatocytes from bile acid-induced apoptosis via activation of survival pathways. *Hepatology* 39 1563–1573. 10.1002/hep.20246 15185297

[B14] Seyhun E. Malo A. Schafer C. Moskaluk C. A. Hoffmann R. T. Goke B. (2011). Tauroursodeoxycholic acid reduces endoplasmic reticulum stress, acinar cell damage, and systemic inflammation in acute pancreatitis. *Am. J. Physiol. Gastrointest. Liver Physiol.* 301 G773–G782. 21778463 10.1152/ajpgi.00483.2010

[B15] Signoretti M. Roggiolani R. Stornello C. Delle F. G. Capurso G. (2017). Gut microbiota and pancreatic diseases. *Miner. Gastroenterol. Dietol.* 63 399–410. 10.23736/S1121-421X.17.02387-X 28240004

[B16] Song S. Tan J. Miao Y. Sun Z. Zhang Q. (2018). Intermittent-hypoxia-induced autophagy activation through the er-stress-related PERK/eIF2alpha/ATF4 pathway is a protective response to pancreatic beta-cell apoptosis. *Cell. Physiol. Biochem.* 51 2955–2971. 10.1159/000496047 30562747

[B17] Sun L. Xie C. Wang G. Wu Y. Wu Q. (2018). Gut microbiota and intestinal FXR mediate the clinical benefits of metformin. *Nat. Med.* 24 1919–1929. 10.1038/s41591-018-0222-4 30397356 PMC6479226

[B18] Tan C. Ling Z. Huang Y. Cao Y. Liu Q. (2015). Dysbiosis of intestinal microbiota associated with inflammation involved in the progression of acute pancreatitis. *Pancreas* 44 868–875. 10.1097/mpa.0000000000000355 25931253

[B19] Tenner S. Baillie J. DeWitt J. Vege S. S. (2013). American College of Gastroenterology guideline: management of acute pancreatitis. *Am. J. Gastroenterol.* 108 1400–1415. 10.1038/ajg.2013.218 23896955

[B20] Vege S. S. DiMagno M. J. Forsmark C. E. Martel M. Barkun A. N. (2018). Initial medical treatment of acute pancreatitis: american gastroenterological association institute technical review. *Gastroenterology* 154 1103–1139. 10.1053/j.gastro.2018.01.031 29421596

[B21] Wan Y. D. Zhu R. X. Bian Z. Z. Pan X. T. (2019). Improvement of gut microbiota by inhibition of P38 mitogen-activated protein kinase (MAPK) signaling pathway in rats with severe acute pancreatitis. *Med. Sci. Monit.* 25 4609–4616. 10.12659/msm.914538 31226101 PMC6599419

[B22] Yoshimoto S. Loo T. M. Atarashi K. Kanda H. Sato S. (2013). Obesity-induced gut microbial metabolite promotes liver cancer through senescence secretome. *Nature* 499 97–101. 10.1038/nature12347 23803760

[B23] Zheng J. Lou L. Fan J. Huang C. Mei Q. (2019). Commensal *Escherichia coli* aggravates acute necrotizing pancreatitis through targeting of intestinal epithelial cells. *Appl. Environ. Microbiol.* 85:e00059-19. 10.1128/AEM.00059-19 PMC6544826 30979838

[B24] Zhu Y. He C. Li X. Cai Y. Hu J. (2019). Gut microbiota dysbiosis worsens the severity of acute pancreatitis in patients and mice. *J. Gastroenterol.* 54 347–358. 10.1007/s00535-018-1529-0 30519748

