# Supplementary material for: Bile Acid Supplementation Improves Murine Pancreatitis in Association With the Gut Microbiota
Source: Front Physiol. 2020 Jun 16;11:650. doi: 10.3389/fphys.2020.00650 (PMC7309677; doi:10.3389/fphys.2020.00650)
Supplement: Supplementary file 1 [file Table_1.doc]

SUPPLEMENTAL MATERIAL

**Bile acid supplementation improves** **murine pancreatitis in association with the gut microbiota**

**Supplemental materials and methods**

**Gut microbiota-depleted mice model**

Mice were administered a combination of four antibiotics (ABX): ampicillin, neomycin,metronidazole, and vancomycin (Sangon Biotech, Shanghai, China) by gavage (10 mg/mL, 1 mL/100 g body weight, twice a day) for 21 consecutive days. Afterwards, acute pancreatitis was induced on the 22th day. Fecal samples were harvested before and after antibiotic treatment. Validation of the gut bacterial depletion was performed by a fecal culture, in which brain heart infusion agar (BHIA) (Oxoid Ltd., asingstoke, Hampshire, UK) plates were incubated anaerobically at 37°C for 48 hours.

**Microbiota transplantation mice model**

Stool (20 mg) of acute pancreatitis was dissolved in 1 ml saline, shaken for 3 min, centrifuged for 3 min at 4 °C, and 200μl supernatant collected. Gut microbiota depleted mice were fed 100μl of the above supernatant by gavage for three days.

**16S rDNA gene sequencing**

Briefy, 24 h afer AP induction, ileal segments were opened. The ileal contents were collected and immediately stored at −80 °C for DNA extraction. DNA extraction was proceeded using a DNA isolation kit (MoBio Laboratories Inc.,Carlsbad, USA) following the manufacturers’ instructions. Bacterial DNA was amplified using 338F (5'-ACTCCACGGGAGGCA-3') and 806R (5'-GGACTACHVGGGTWTCT-3') primers covering V3-V4 region of the bacterial 16S rRNA gene. PCR reactions were performed in triplicate 20 µl mixture including 4 µl of FastPfu Buffer, 2 µl of 2.5 mM dNTPs, 0.8μL of each primer (5μM) (Sangon Biotech), 0.4 μl of FastPfu Polymerase (TransStart FastPfu DNA Polymerase, TransGenBioTech, Beijing, China), and 10 ng of DNA template. The PCR products were evaluated using 2% (wt/vol) agarose gel electrophoresis and purified using the AxyPrep DNA Gel Extraction and quantified via QuantiFluor-ST (Promega, Madison, USA). Equimolar concentrations of amplifications were pooled and sequenced on an Illumina MiSeq platform (Illumina, San Diego, CA, USA) according to the manufacturer's protocol. Data were analyzed using QIIME and Mothur software.

**Intestinal microbiota Functional annotation**

The metagenomes of gut microbiome were imputed from16S rRNA sequences with Phylogenetic Investigation of Communities by Reconstruction of Unobserved States (PICRUSt). This method predicts the gene family abundance from the phylogenetic information with an estimated accuracy of 0.8. The closed OTU table was used as the input for metagenome imputation and was first rarefied to an even sequencing depth prior to the PICRUSt analysis. Next, the resulting OTU table was normalized by 16S rRNA gene copy number. The gene content was predicted for each individual. Then the predicted functional composition profiles were collapsed into level 3 of KEGG database pathways.

**Bile acid analysis**

The BA levels in feces were quantitatively measured by ultra-performance liquid chromatography triple quadrupole mass spectrometry (UPLC-TQMS) according to a protocol.The feces samples were extracted with methanol and the supernatant were transferred and vacuum-dried. After reconstituted with mobile phase, the serum extract as well as the bile acid reference standards were analyzed with a Waters ACQUITY ultra performance liquid chromatography coupled with a Waters XEVO TQ-S mass spectrometer with an ESI source (Waters, Milford, MA). The entire UPLC–MS/MS system was controlled by MassLynx 4.1 software. All chromatographic separations were performed with an ACQUITY BEH C18 column (1.7 µm, 100 mm x 2.1 mm internal dimensions) (Waters, Milford, MA) and the injection volume was 5 µL. UPLC-MS raw data obtained with negative mode were analyzed using TargetLynx applications manager version 4.1 (Waters Corp., Milford, MA) to obtain calibration equations and the quantitative concentration of each bile acid in the samples.

**Sequences of the real time PCR primers**

Fxr: TGGGCTCCGAATCCTCTTAGA(Forward) TGGTCCTCAAATAAGATCCTT GG(Reverse)

Shp:TCTGCAGGTCGTCCGACTATTC(Forward) AGGCAGTGGCTGTGAGAT GC(Reverse)

Fgf15: GCCATCAAGGACGTCAGCA(Forward) CTTCCTCCGAGTAGCGAATC AG(Reverse)
