# Supplementary figures and images for: Bile Acid Supplementation Improves Murine Pancreatitis in Association With the Gut Microbiota
Source: Front Physiol. 2020 Jun 16;11:650. doi: 10.3389/fphys.2020.00650 (PMC7309677; doi:10.3389/fphys.2020.00650)

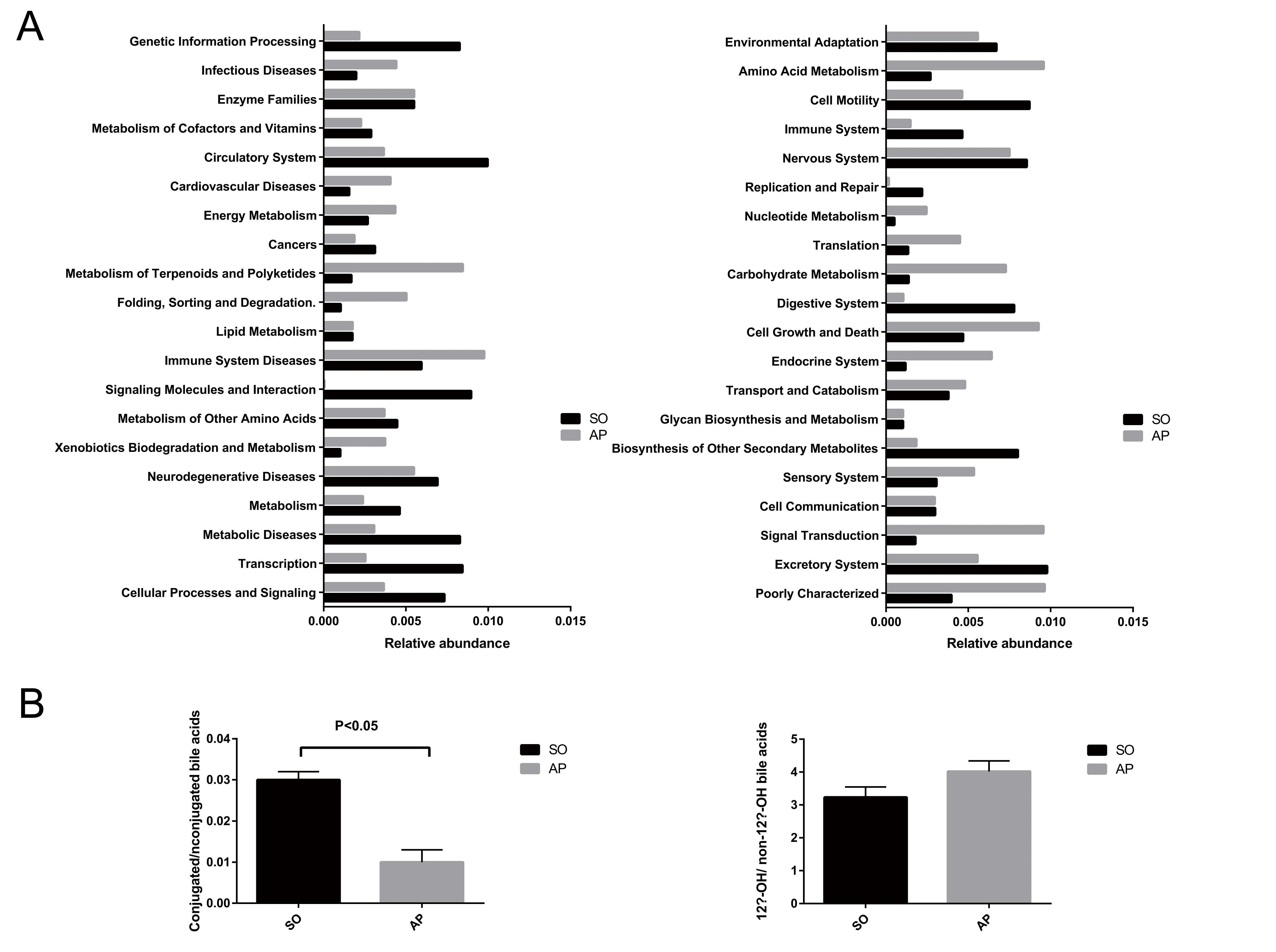

Supplement: Supplementary file 2 [file Image_1.TIF]

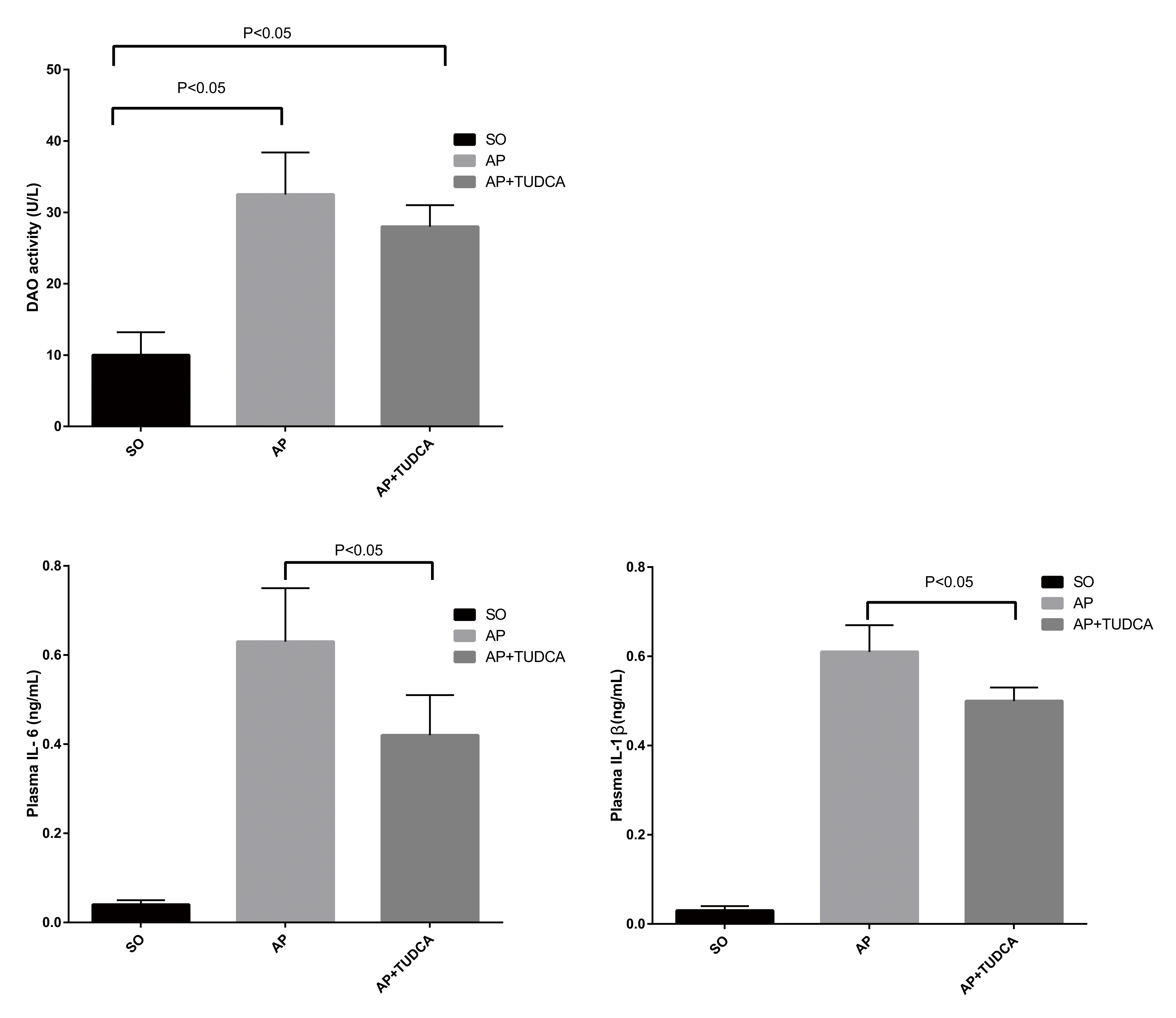

Supplement: Supplementary file 3 [file Image_2.TIF]

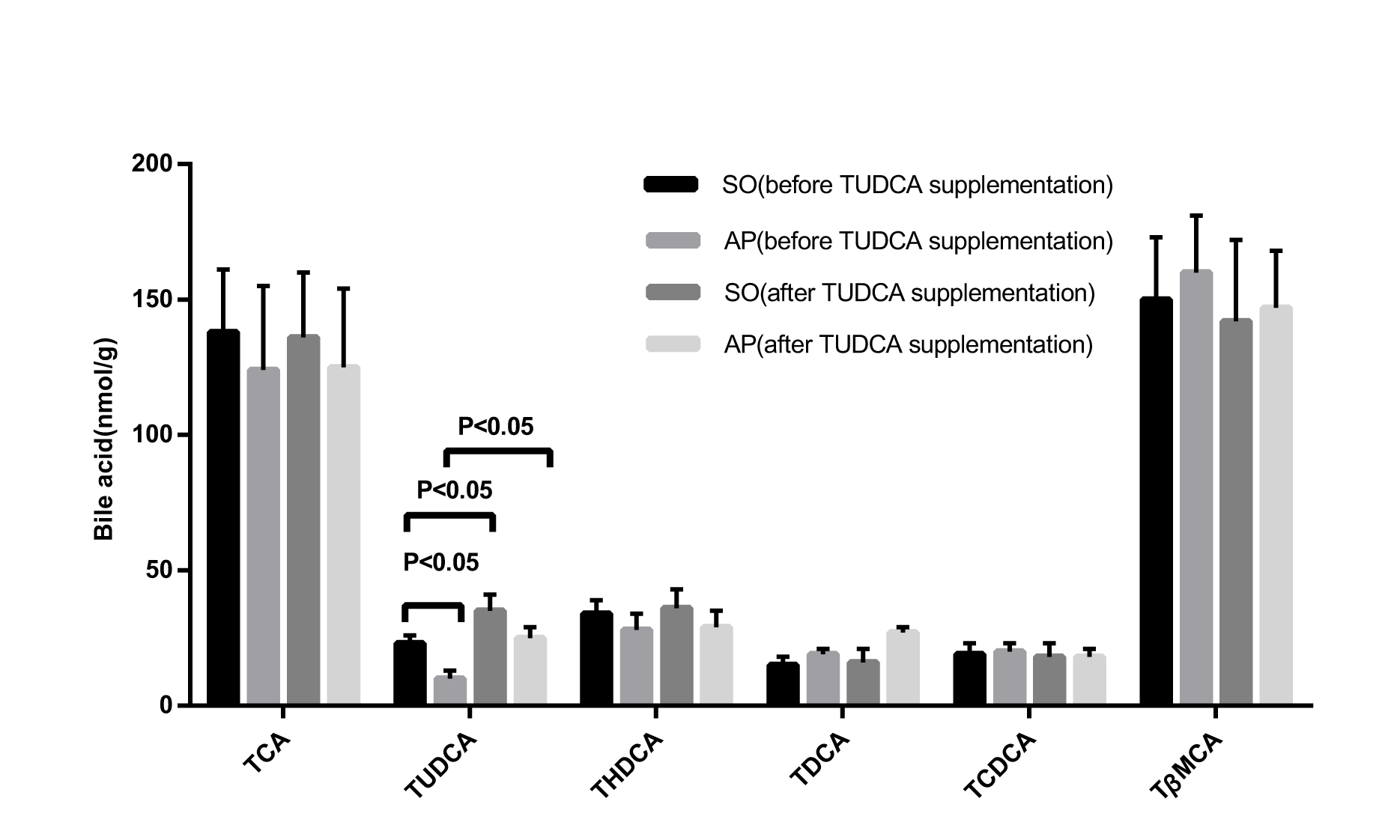

Supplement: Supplementary file 4 [file Image_3.TIF]
